# ‘In the picture’: perspectives on living and working with cancer

**DOI:** 10.1136/medhum-2022-012392

**Published:** 2022-08-04

**Authors:** Sophie Day, Kelly Gleason, Celia Lury, Di Sherlock, William Viney, Helen Ward

**Affiliations:** 1 Anthropology, Goldsmiths University of London, London, UK; 2 Infectious Disease Epidemiology, Imperial College London School of Public Health, London, UK; 3 CRUK Centre, Imperial College London Faculty of Medicine, London, UK; 4 Centre for Interdisciplinary Methodologies, University of Warwick, Coventry, UK; 5 writer, London, UK

**Keywords:** Cancer care, poetry, Medical humanities, social anthropology

## Abstract

We explored working and living with cancer at a large research-intensive National Health Service hospital breast cancer service and adjoining non-governmental organisation (NGO). The project had three elements that were largely autonomous in practice but conceptually integrated through a focus on personalised cancer medicine. Di Sherlock held conversations with staff and patients from which she produced a collection of poems, *Written Portraits*. At the same time, we conducted interviews and observation in the hospital, and hosted a public series of science cafés in the NGO. The trajectory of this project was not predetermined, but we found that the poetry residency provided a context for viewing participation in experimental cancer care and vice versa. Taking themes from the poetry practice, we show how they revealed categories of relevance to participants and illuminated others that circulated in the hospital and NGO. Reciprocally, turning to findings from long-term ethnographic research with patients, we show that their observations were not only representations but also tools for navigating life in waiting with cancer. The categories that we discovered and assembled about living and working with cancer do not readily combine into an encompassing picture, we argue, but instead provide alternating perspectives. Through analysis of different forms of research participation, we hope to contribute to an understanding of how categories are made, recognised and inhabited through situated comparisons. In personalised medicine, category-making is enabled if not dependent on increasingly intensive computation and so the practices seem far removed from mundane processes of interaction. Yet, we emphasise connections with everyday practices, in which people categorise themselves and others routinely according to what they like and resemble.

## Introduction

We explored developments in cancer care through three strands of research from 2018 to 2022. We observed Charing Cross Hospital’s breast cancer services and interviewed staff and patients (Sophie Day, anthropologist and William Viney, medical humanities), aiming to update findings from an earlier study in 2013–2014 (Day *et al*. 2017). Medical care today depends on patients’ contributions to research including formal donations to large data and tissue collections as well as data from routine monitoring of their condition. Patients therefore necessarily participate in experimental or personalised breast cancer care as research subjects even if they do not formally join research studies (Cambrosio *et al.* 2018). Continuing analysis of these data enables cancers to be categorised more precisely through their similarities and through their affinities with available treatments. In this way, it is hoped that outcomes can be improved. We also hosted six public science cafés on personalised cancer medicine (2018–2019) to discuss developments and explore further the positions of staff and patients (Kelly Gleason, Imperial CRUK Lead Nurse, and Day). These cafés were held at Maggie’s West London, a non-governmental organisation (NGO) associated closely with the hospital cancer service. Finally, we introduced a poetry residency with Di Sherlock (2019–2020), hoping that it would offer an avenue for understanding if and how interlocutors related cancers to other aspects of their lives, which was difficult to assess in the hospital and science cafés when cancer was the agreed focus (Fraser and al Sayah 2011; Stuckey and Nobel 2010). We aimed to illuminate different facets of participating in research on personalisation. However, the trajectory of our project was not predetermined and, although we liaised closely across the three project strands, we followed situations that emerged and unfolded independently in each strand of research.

We do not intend to suggest that these components can be combined through addition to give a fuller picture but rather that one perspective places another at a different angle—as though it were approached in different lighting or for different purposes. We foreground the poetry practice in presenting our research collaboration, and use the poetry collection, *Written Portraits* (Sherlock 2020), to introduce our other investigations and some of the findings. The residency spanned Charing Cross Hospital, where Sherlock met staff, and the adjoining NGO, Maggie’s West London, where she met patients. We crossed these two sites routinely with patients and staff, many of whom accepted invitations to join other research as they participated in cancer care. We compare the affinities that Di Sherlock registered in her ‘sitters’—those who joined conversations from which she composed the poems—with the affinities registered in cancer care and show how perspectives shift when data are viewed from the perspective of cancers, various staff, patients or hospital routines to make the most relevant associations visible. These perspectives are related, we suggest, as views are exchanged.

After exploring references to (being) ‘in the picture’ in *Written Portraits*, we take the image of fractals in one of the poems to explore from the perspective of long-standing patients, including the sitter for that poem, who we interviewed repeatedly over a period of years. We re-present the fractal ‘portrait’ as a *route* that patients take through multiple interactions and materials that combine and collide in the hospital and beyond. In this perspective, patients position themselves in fractal relations that do not only represent but compare and evaluate life in waiting. This summary from our ethnographic research provides a commentary on Sherlock’s *Written Portraits* as well as participation in cancer care.

We ask how the poetry residency illuminates and is illuminated by participation in personalised cancer care. Despite evident and striking differences between the art practice, public discussions about cancer and cancer care, we hope to contribute to discussions in the medical humanities by showing how each can become a context or foil for the other. Participants in the residency—as in other research—sometimes found the experience therapeutic and engaging; they valued seeing themselves through another’s literary representation (Boydell *et al*. 2012; Henare, Hocking, and Smythe 2016; Majid and Kandasamy 2021; Tarr, Cornish, and Gonzalez-Polledo 2018). Poetry, in this context, is not a luxury (Lorde(Lorde 1984) 1977/1984) but, in Lorde’s view, a distillation of experience that illuminates ‘connections between self- and collective transformations’ (Ferguson 2012, 296). Here, however, we restrict our discussion to aspects of personalisation that emerged through the hospital ethnography, the public science cafés and the poetry residency, all of which occurred during the same period, at the same sites and with some of the same people. Exploring the representations formed from registering different kinds of participation, we ‘lead’ from different strands of our research. Initially, we foreground the poetry residency; subsequently, we present an alternative perspective from ethnographic research with patients on fractals.

This sense of perspective was framed helpfully in a subsequent discussion with students in 2021. We introduced *Written Portraits* to a class on participatory approaches in health research, and a student asked on the zoom chat, ‘who’s leading?’. The class suggested—with reference to cancer as a dance partner in the poem, ‘An Occasional Inconvenience’—that Di Sherlock, cancers and people living and working with cancer were all leading in different ways and at different times. Cancers, sitters and the poet might take turns, they thought, according to implicit and situational rules akin to turn-taking in conversations (Ochs and Capps 2009). The dance could be a *pas de deux* or it might, as the next contributor explained, ‘vary with the tune’. Who or what was leading, the class decided, depended on circumstance and happenstance, as summarised through the chat function in a concluding comment, ‘again the perspective will decide who leads!’.1 We imagined that the three strands of our research might remain distinct, as they did in key respects, but we hope that they also suggest novel insights into how categories are made, recognised and inhabited through situated comparisons. Our analysis responds to an invitation for a critical medical humanities (Viney, Callard, and Woods 2015), which intervenes in and reorders what Des Fitzgerald and Felicity Callard have called the favoured topoi of medical humanities scholarship (Fitzgerald and Callard 2016).2

## Methods

### Patient and public involvement

Patients and staff were involved in the design, conduct, reporting and dissemination of our research. The Imperial Public Involvement Group for Cancer commented on the initial design, which was amended to incorporate their feedback. Representatives from the hospital cancer services, Maggie’s Centres and a four-member patient advisory group have guided the project, 2018–2022.

Patients and staff participated in all three strands of the research we describe. Observations and interviews in the breast cancer service mirrored our earlier study in 2013–2014.3 The science cafés attracted audiences of up to 35 scientists, healthcare staff, patients, family and friends to lively discussions about cancer as well as a panel discussion about implementing personalised approaches in local National Health Service (NHS). We took notes on discussions, collected anonymous feedback and conducted interviews with some participants.

Once we were given research permission, Sherlock was able to invite participants to share their stories with her. She planned to explore how individuals and small groups established resemblances and affinities with other people, objects, imagery and environments. During open conversation(s), Di Sherlock made handwritten notes and later wrote a poem based on the notes and her recall of the sitter and the conversation. The poem was given back to sitter/s who were invited to edit it with Sherlock and reaffirm consent to their participation in writing. This practice sometimes involved two meetings: an initial conversation from which a poem was crafted and a second conversation to collaborate in finishing it. Other poems unfolded over a series of conversations with several individuals and involved extensive to-and-fro before sitters liked how they were depicted and voiced, and Sherlock liked how the poem read and sounded.

Participants were eligible to join all three of our enquiries, and some did. We sought connections alongside evident contrasts between them to ask how people ‘do’ participation, to what ends and over what time spans. Recognising the radical differences between what is understood to constitute data and data ethics (Shildrick *et al*. 2018), we nevertheless asked how dialogues between researchers, staff and/or patients were registered as data and influenced by the invitation to participate (Moniz *et al.* 2021; Tarr, Gonzalez-Polledo, and Cornish 2017).

Di Sherlock described ‘the transaction’ of a conversation for a poem that would elicit written consent within days or weeks. She assessed a portrait’s resemblance with a sitter through their response: if a sitter liked their poem, its likeness was established. The residency thus deployed what Nicolas Bourriaud called relational aesthetics to describe art based in or inspired by social relations and their contexts and exchanged with viewers or readers (Bourriaud 1998). Sherlock hoped that her portraits would be recognisable and introduce a new perspective—how sitters saw themselves in the way the poet saw them. She explained how she wrote poems one at a time in a ‘zone’ in the hope that voices would emerge in dialogue and her own would remain situated alongside others rather than a proxy, appropriation or impersonation (Tomlin 2013). She worried about fictionalising an account when she was left ‘dangling’, unable to finish a conversation or return a poem to her sitters. Poems also had ‘to be readable’ and are suited to reading aloud: Sherlock’s use of the vernacular is a collage of verbatim and references to relations, objects, events, and imagery within and beyond the looks and words exchanged between herself and sitters.

Sherlock explained that there would be no point to her practice if the sitter did not like their poem: ‘it would be a dud’, she said. She shared drafts to check for accuracy and sitters suggested changes. Some staff found the material unexpectedly and, in some cases, uncomfortably personal while patients, in contrast, wanted to add details. Brief commentaries distilled from research analysis by Di Sherlock and Sophie Day during 2020 on Zoom reflect some of these adjustments in a digital commentary, ‘About this Poem’. They show how participants from both Maggie’s and the hospital carefully removed or, after consultation, approved reference to others who were important in their lives but absent from conversations with the poet. A nurse requested that Sherlock make references to her family ‘more generic’, while a patient described negotiations with her sister who insisted on removing family references that she found intrusive, and the poem was re-drafted. Redaction on the part of sitters thus censored relations that made up the portrait.4

Edited, this rendering of a limited time spent together in Maggie’s or the hospital concluded when liking and likeness coincided. Subsequently, Sherlock distinguished relations to finished portraits, considering herself author of a poem that she co-owned with sitters. It was up to sitters whether they shared their poem but, in this residency, they not only sent them to family and friends, and shared them on social media, but also all agreed to publication online and in a book. *Written Portraits* includes six ‘group portraits’ involving several individuals and 20 ‘individual portraits.’

## Written portraits

The poetry collection has two parts which introduce and connect the main research settings of Charing Cross Hospital and Maggie’s West London. In presenting the collection, we refer to what we were learning at the same time and from some of the same participants in the science cafés and the hospital’s breast cancer services. This juxtaposition establishes the grounds for our subsequent discussion about making categories on the basis of similarities and affinities.

‘On First Visiting Maggie’s West’ is the first poem of the first part of the collection. We learn that Maggie’s is,

In the precinct of the Hospital

outside its jurisdiction

an Orange Box –

tangerine puzzle between worlds

roof in flight

entrance hidden …

The Box unfolds, an origami of light.

The fickle spring sky is everywhere.

Rainbows glimmer on the wood as the busy kettle serves

The Kitchen Table - the hub, the nub, the agora

Maggie’s Centres are designed by internationally renowned architects to reflect both interdependence with hospital care and a distinctive healing architecture. Charles Jencks coined the term kitchenism to reflect the importance of hospitality and the domestic to the brand (Jencks and Heathcote 2015). Visiting Maggie’s West London is voluntary, and visitors can position themselves inside and outside the building, its activities, and services by moving from the central table to a meeting with a member of staff or one of the several gardens and corners. Several Maggie’s staff, including the Centre Head, worked previously in the adjoining hospital, and psychological support for those living with cancer effectively straddles the two organisations.

The portraits in this part of the collection, ‘Round the Kitchen Table’, were shaped by the times and spaces afforded by the table. Sherlock was invited to just turn up and—with permission from those already present—re-introduce her practice, sit and listen: ‘Positions around the table take account of who’s there and who isn’t in relation to absences as well as presences, and preferences, which shift. Over several weeks, the group appears through shifting patterns rather like time lapse photography. … [T]he pattern of the group emerges here through the rhythms of the poem’ (‘Knights of the Oblong Table’). A home away from home, it was easier to join the table at Maggie’s as a guest than to draw conversation to an end as a host and leave.

Sherlock notes how an elusive sitter cut short his sitting to help organise the Science Café that evening, which took place in a large space near the central kitchen. It was one of many occasions at Maggie’s when participants tried to understand their position in hospital routines: ‘The Three Musketeers’ opens at Maggie’s kitchen table and explains how two speakers met in the hospital biopsy queue; ‘Oncology’ says the sitter in ‘Howling Wolf’, explaining why she turned up an hour late for conversation with Sherlock. At the science café, it was the texture of treatment pathways through the hospital that interested participants. This was our sixth and final event involving a panel with a clinical oncologist, a senior oncology nurse, two patients, one of whom had worked as a nurse, and a complementary therapist. Most of the other participants (‘audience’) were people living with cancer along with a few staff and companions. One patient on the panel described the pathway as a conveyor belt and another reflected on her perspective as both nurse and patient. What had appeared as orderly patient tracking lists and mandated timelines when she worked as a nurse now felt like a whirlwind rush. She had no time to process information in the main oncology clinic where she was one of ‘a huge mass’ that seemed ‘like a city’. The early stages involved information, the quantity of which another patient had misjudged when he took the first batch as a ‘two-pint problem’ to read for an hour or so in the pub. He explained that feeling like an item on a conveyor belt resolved with time since ‘the personalised does come through after the early rush. It disappears gradually as treatment carries on’. Others described how hard it was to step off this conveyor belt as involvement with services lessened or disappeared. Like an item on a conveyor belt, you had been passed from one point to another in what was also glossed—from the position of that item—as a ‘relay race’. Then, suddenly, you are ‘all clear’. The nurse and oncologist on the panel agreed that personalised medicine was leading to more complex diagnoses, more patients and more steps to treatment.

Hospital Corners is the name of the second part of *Written Portraits* and ‘The Hospital Tree’ is its first poem. The hospital is represented through its clock, which had stopped. Images from Dante’s Divine Comedy in the poem suggest purgatory for patients and visitors who wait in this uncertain and unreliable clock time, figured against a background of exceedingly busy staff, ‘a constant traffic unremarked’, who protect themselves from conversation by evident hurry, clipboards and trolleys as they connect and provision the many parts of the building.

Staff and patients navigated several further desks, kiosks and queues once they were past the main reception’s revolving door. Cancer services are found upstairs and along corridors; patients check in, and many have their bloods taken, wait for clinical appointments and for prescription medicines over the course of one or two visits. Mysterious titles and names appear in *Written Portraits*—Clinic 8, East 6, West 6, the doctor, metastatic breast cancer specialist nurse, senior sister, matron, ward sister, manager, unit manager—associated with different points of the treatment pathway for patients and different skills or roles among staff. A reader might sense that it is difficult to find your way initially from one person or place to another and, inside the building, signs only occasionally make parts of the pathway visible.

Personalised cancer medicine is delivered in what Sherlock represents as a patchy real estate with leaking roofs and power outages amid countless staff, machines, cafés, waiting rooms and lines of different colours that disappear into the distance. It is embedded in historical legacies of race and empire, as well as older understandings of cancer. In our hospital ethnography, we found that more categories of breast cancer were defined by staff than 5 years previously and some were associated with different treatments and prognoses, based largely on molecular characterisation. Oncologists and other staff tried to establish how particular cancers resembled or differed from others in order to match them with available treatments and avoid or delay negative sequelae. As an oncologist concluded, summarising developments over their career during an interview: ‘Twenty-five years ago each type of cancer had two, two or three, types of treatment but now, each has got ten types’.

Sitters (staff and patients) participated in both cancer care and the poetry residency as experimental subjects. But Sherlock’s ability to talk and listen to sitters in the hospital was severely constrained. She had to seize moments of conversation in ‘hospital corners’ at the beginning and end of shifts. Instead of struggling to extricate herself as at Maggie’s kitchen table, she found that she was put on hold in the hospital and delayed by competing priorities, demands and hierarchies.

Professional roles among clinical staff in hospital corners made their personal experiences largely invisible to other people, but the poems show them travelling, creating ties of adoptive kinship with co-workers, and living with or around cancers at home. A volunteer speaks of her own diagnosis. An oncology Registrar is seen through the poet’s eyes working at Charing Cross after his father’s cancer treatment in Birmingham; he is both doctor and son. Several poems also describe combinations of research and care. The first conversation of the residency was arranged with three members of Gleason’s team and reported in ‘At the Bottom of the Pyramid’. The three are presented individually and as they manage studies within the NHS along with an absent fourth. They are called journey-women, a reference that expresses both the historical sense of skilled workers who have completed their apprenticeship and the care with which they accompanied other staff and patients so that, in assisting others’ journeys, they embarked on journeys of their own. Research technicians also took care of data and tissue which travelled on still further routes through research studies that produced results which might help update care pathways for subsequent patients. ‘More Than One Life’ portrays research in one of the laboratories and ‘The Art of Medicine’ describes uneasy combinations of research and care in the drugs a doctor-scientist used, which caused devastation as well as healing. *Written Portraits* suggests resemblances and affinities as well as exchanges between patients in the first part of the collection and staff in the second.

## Making associations and categories

Building on annotations in the online version of *Written Portraits,* we present fleeting, provisional and more stable categories of association established on the basis of what people liked and who they were like. The phrase ‘People Like You’, the name of our collaborative project (see https://peoplelikeyou.ac.uk), refers to the personalised address that is familiar from Amazon, Spotify or Netflix marketing: ‘people like you like things like this’. Practices of ‘liking’ (affinity or preference) and ‘likeness’ (resemblance or similarity) are varied and they inter-relate (Bourdieu 1984). In *Written Portraits*, sitters were portrayed through what they liked, qualities that resonated with the poet, and names, references or attributes that people shared, including types of cancer. Florence (‘Everyday Heroines’), for example, may suggest Florence Nightingale to a reader, the lady of the lamp from an earlier imperial workforce, but we learn that the name is intended for a relative in a contemporary west African lineage. In ‘French Connection’, the sitter explains that patients say she shares a name with Winston Churchill’s wife, but the poem indicates that she herself favours North African connections epitomised in the fruit named after monk gardener, Frère Clément. ‘The Art of Medicine’ draws poet and sitter into a common likeness as artists before referring to another kind of ‘people like us’, namely doctors, that excludes the poet. In group poems such as ‘The Three Musketeers’, two men belong together because they both have prostate cancer, but the protagonists also differentiate themselves through previous sparring as Mods and Rockers and through their relative positions, also competitive, in the hospital queue noted above.

The making of a picture is elaborated in three poems. The rhythm of lists or resonant sounds, the layout of words on a page, descriptions of finding your way into or around a building and the layering of past experiences into the present through a gesture all help assemble this picture by shifting the narrative from one association to another. ‘In the Picture’ presents Malcolm talking about his life through the pictures stored on his phone. He puts himself into four categories, two of which were presented as images. First, he saw himself in situ, that is, ‘Round the Kitchen Table’, where he had been invited to join the project *Life*, a collection of portraits of visitors to Maggie’s by Zoe Law.5 He joined the UK book tour, speaking at various venues and shared an image of himself in *Written Portraits* standing in front of his supersize blown up image advertising the book launch. Another invitation came through an advertisement in *The Sun* newspaper. Readers were invited to apply to a competition in which successful candidates would join a group of 70-year-old people and HRH Prince Charles on the stairs at Spencer House for a portrait. Malcolm was successful and showed Sherlock the picture that was subsequently published. Malcolm also placed himself among colleagues at work in the aviation industry and as a man born in India.

Together with relations to family and his childhood home that he recalled in Maggie’s West London, Malcolm enabled Sherlock to represent him through his circumstances, resemblances and affinities. He belonged to a WhatsApp group for a choir that initially met at Maggie’s. Members kept in touch during lockdown in 2020 and at least one reacted to Malcolm’s uncharacteristic silence with what she told Day later was a sense of foreboding. Visitors to Maggie’s anticipated the loss of companions and acknowledged deaths, often obliquely, when they met online or off. This woman later commented on the bittersweet presence of an absent Malcolm with his dapper dress code and inviting manner in his ‘written portrait’: ‘In the Picture’ concludes with Malcolm’s observation of his *Life* portrait, ‘When I’m gone it’ll be here’, as indeed it was.

If Malcolm pictured his past and present life with his phone, two other poems describe how sitters insisted on putting the poet ‘in the picture’ so that she could make a fitting portrait. The sitter in ‘Howling Wolf’ was concerned about the accurate representation of her treatment with chemotherapy, targeted therapies and surgery from the first signs in 2014 to the most recent events of 2019. This history is conveyed in crisp, clear verses interspersed with records of conversations in which the sitter checks the poet’s notes. The sitter insists on ‘correct’ punctuation which would not leave a reader the interpretive flexibility intended by Sherlock. A verse separates the account of cancer treatment from the poet’s reflections,

Satisfied I am now

properly in the picture,

she closes her diary.

Sherlock is *put* in the picture as she recognises and records her sitter’s views.

Similarly, in the science café discussion described above, different positions were acknowledged in exchanges between panel and audience. A range of individuals described how they were identified and affiliated to categories circulating within and beyond the hospital. During a discussion of health professionals’ responses to patients, a woman in the audience was prompted to ask, ‘what about patients?’. She wondered about ‘good’ and ‘bad’ patient behaviour that might help explain staff responses. A member of staff in the audience expressed her difficulties in responding to people who ‘didn’t think you looked the part’. A moving exchange followed as a man, a patient in the hospital, described visiting his local bank where he was mistaken for a member of staff. Everyone agreed that you could seem too young—‘the age of my children or grandchildren’—the wrong gender or skin colour. It became easier for patients and staff to reflect on what they held in common than when they met along treatment pathways. Delicately acknowledging discriminating stereotypes, patients saw themselves through staff seeing them and vice versa, especially when this man laughed about being the sort of person who would be employed in a bank—the ‘right’ age, gender and ethnicity, and with the ‘right’ demeanour. These views of appropriate behaviour privilege a propriety that includes and excludes different people (Fitzgerald et al. 2020), and as perspectives were exchanged in science cafés and the poetry residency, such views were both acknowledged and contested.

The third poem, ‘Per Ardua ad Astra’, sequences and embeds relations of likeness and liking in the sitter’s references and citations to family members, living and dead, through which she adopts their position to speak of herself. Relationships with her father and mother together with their several texts and artworks are layered into the patterning of illness, work and travel. Sherlock considers that Suzanne, the sitter, weaves a view of herself as a fractal person through attending to this self-similarity. Again, the poet is put in the picture, but Sherlock explains that it is she who is now putting herself in the picture of her sitter and ‘addressing self, sitter and reader(s) equally’ in a collage of the fractal ‘past (the noted/remembered conversation), “the other” (sitter), the present (the act of writing in the park) and the “I” (the writer)’.

The conversation takes an unexpected turn

and I’m doing the talking,

recalling my journey through

my mother’s dementia and the cancers

that took my father and brother.

The pastoral gaze is clear, penetrant,

the eyes infinitely kind.

These fractal patterns take a narrative form in place of more familiar visual geometries. Shifting from one category to another is an implicit or explicit ‘like’, which is linked to the next through a turn in the conversation or a thread chosen by the author. The same and not the same, a comparison that pivots a resemblance or affinity so that it is at one moment external and the next integral to the person. The anthropologist Roy Wagner might have been commenting on this portrait of Suzanne when he observed, ‘A fractal person is never a unit standing in relation to an aggregate, or an aggregate standing in relation to a unit, but always an entity with relationship integrally implied’ (Wagner 1991, 163). *Written Portraits* raises questions, therefore, about the ‘unique’ individuals portrayed. If these are fractals, they are not the individuals of liberal ideologies who exist independently from society (Strathern 1991). The patterning identifies a range of people, including the poet, cultural references, histories and environments through which a picture emerges.

Associations that are registered depend on what is pertinent, as noted in the online class mentioned above (Introduction). It is cancers that lead and require contextualisation from clinicians in the outpatients’ department. Patients and staff recognised that cancers and persons are intermeshed and, during clinical consultations that we observed, the selective effects of treatment on one ‘dance partner’ rather than the other were assessed. As hosts for cancers, patients understood that they would embody signs that oncologists with their tools might be able to decipher. We found that approximate associations between ‘liking’ and ‘likeness’ emerged gradually and uncertainly in response to interpretations of imaging and biopsies; other forms of measuring, counting and calculating; examinations that drew on professional judgement and statistical analysis; trial and error and new research evidence from consortia of laboratories and hospitals that were introduced periodically to the service. As an oncologist summarised, ‘There are, no more averages. I can quote averages from clinical trials, but we don’t know. … [a test] may come back with lots of mutations that there’s no drug for, or there may be several mutations. Which mutation do we go to first? If there’s a drug, it’s not available or they may have already had some of that treatment and there may be heterogeneity within a tumour. If we biopsied a different part of that tumour or a different metastasis, that might throw up different results.’ (cited in Day et al. 2021)

Oncologists also explained difficulties in predicting how cancers might change through, for example, developing resistance to therapies. At one science café, a laboratory researcher presented breast cancer as a ‘paradigm disease’ for Precision (or Personalised) Medicine with reference to a particular type called HER2-positive cancer. She explained that everyone ‘has a different cancer… a different somatic and copy number change’ but the group’s statisticians ‘optimised’ findings so as ‘to avoid noise’ and assess the utility of different markers for screening and detecting recurrence in clinical settings. The precision of what she called ‘personalised blood tests’, which revealed every cancer to be unique, had to be balanced against statistical and other generalisations, which asked what differences matter. In these exchanges, as in the poems, resemblances and affinities are recorded that are relevant to the task at hand.

Interviews illustrated the quandaries for patients as they discussed HER2-positive cancers. When a woman who had been living with cancer for over 10 years found her cancer re-categorised with a HER2-positive diagnosis, she was recommended a new treatment plan and found herself, ‘emotionally, you‘re in this, sort of, bubble of a routine where you know that you‘re going to get your little tablets and that becomes your safety net. And then all of a sudden, you‘re now going to expose yourself to another path. … so I went along, met with Dr [X] again, after the biopsy, and she told me she would like me to have a course of chemotherapy and that it would go alongside the antibodies, Herceptin (a brand name for trastuzumab), and I think another one, which I don't remember the name. That I would be going in three weekly.’ Another woman referred to the treatment, trastuzumab, because she was worried about her poor prognosis and thought that medical research into cancer types was ‘all good so long as it doesn’t mean that I can’t have something like Herceptin in a year’s time when everything else has run out, if that would give me another six months’ (cited in Day et al. 2021). Trastuzumab was not used to target the cancer that had been established some time previously as her ‘dance partner’ but this woman was as aware as her oncologist of the dynamic heterogeneity of cancers. She thought another biopsy might establish changes in her tumours’ resemblances and affinities and, if they had become partially HER2-positive, she could look forward to the possibility of effective treatment.

## Patients’ perspectives

Di Sherlock directed open conversations over a short period of time into ‘written portraits’ but biological, data and clinical perspectives are coordinated for their relevance in guiding treatment and understanding over an indefinite period. During months or years of participation, patients offered multiple views on living with cancer. Repeated observations and interviews with a few long-standing patients attending breast cancer services between 2013 and 2020 showed how women strung names, categories and events together in varied ways. Inspired by Susan Gal’s analysis of the public/private distinction, we suggest that patients navigated their lives in waiting (Day 2015).

Gal shows that distinctions between public and private are partially aligned in what she calls recursive fractals; what the terms indicate differ in each application ‘while deceptively retaining the same label and the same co-constituting contrast’ (Gal 2002, 83).

Whatever the local, historically specific content of the dichotomy, the distinction between public and private can be reproduced repeatedly by projecting it onto narrower contexts or broader ones. Or, it can be projected onto different social “objects” – activities, identities, institutions, spaces and interactions – that can be further categorized into private and public parts. Then, through recursivity (and recalibration), each of these parts can be recategorized again, by the same public/private distinction. (Gal 2002, 81)

Waiting *for* and *with* treatments, cancers and other people in Charing Cross Hospital and Maggie’s West London, women implicitly or explicitly evaluated their position in an opaque and shape-shifting collective. We summarise key themes to illustrate a sense of fractal relations that unfolded and positioned participants over time. We hope this ethnography extends our account of being ‘in the picture’ in *Written Portraits*.

Initial experiences after diagnosis of waiting on ‘a conveyor belt’ in the hospital indicated bureaucratic indifference to supplicants alongside evident hospital efforts and intricate choreographies to apply rules or procedures fairly. As ‘Howling Wolf’ showed, patients with cancer expected to sit or stand for an hour or two after their scheduled appointment time, wait weeks for results and months for evaluation of the effects of a therapy. Perspective is seemingly erased by hospital standards that integrate features measured on varied dimensions into a single—as it were objective—scale of commensuration. Enrolled under uniform standards like those mentioned at the science café panel to track patients through the mandated times between points on a pathway, women put themselves inside this view from nowhere. They associated their own cancers uncertainly with those attached to people waiting alongside in the hospital and ‘Round the Kitchen Table’. They tried to establish how their cancer was both similar to and distinct from another, as illustrated in an interview describing how a woman became friends with another patient. The woman explained in her interview, ‘we were in the same room in the hospital when we had the operation. So that’s when we became friends.’. Happening to spend time together, they compared experiences. Subsequently, however, their paths through care diverged, and this woman found it difficult to explain her inferences to her friend. She said that she told her friend, ‘your diagnosis could be different to mine’, but her friend rejected this suggestion by pointing again to features that they shared, ‘no, we’ve got a stage three’. The speaker insisted, ‘I said, “It’s probably the type of cancer we have. Yours is different to mine.”’

Patients found it difficult to apply and relate categories of cancer medicine to their lives but, as Susan Gal emphasises, people inevitably develop a view from somewhere and take up positions inside as well as outside their observations (Gal 2016; see also Gal and Irvine 2019; Irvine and Gal 2000). Patients noticed and responded to each other. They dealt with an overwhelming sense of assembly line care by contrasting, calibrating and interrelating different features of waiting. Waiting *for* NHS procedures as opposed to waiting *with* people in shared circumstances constituted a way to construct points of view. These few women led category-making by differentiating, evaluating, and reapplying practices of waiting to other situations, such as formal research.

Patients are encouraged if not expected to participate in hospital research. Those that join formal studies anticipate better care than the usual in return (Day et al. 2021; McGrath-Lone *et al*. 2016; Viney et al. 2022). In addition to a direct transaction of research participation for care (insofar as they can be distinguished), women expressed a commitment to research that is often described as altruism, care for possible futures and for others (Puig de La Bellacasa 2017). Long-standing patients attending the hospital bridged established and evolving understandings of cancer in the hope of eventual improvement in outcomes as they developed social lives around the hospital service. Joining a choir, a Nordic walking group or the kitchen table that they found at Maggie’s West London, they became friendly with staff such as the research technicians or ourselves, waiting with them for instructions or results in the service. For example, after participating in two of the three enquiries we have described, Jayne Smith summarised various discussions with Day and Ward (university staff) in an email (2019),

From almost the beginning of my treatment I became involved in some kind of research. That, in itself, gave me some purpose in dealing with my disease, with a hope that my misfortune could eventually be beneficial to other breast cancer sufferers, and it therefore put a positive spin on my condition. … (cited in Day, Smith, and Ward in press)

Other women expressed a similar commitment to participating in experimental cancer care. Most had been attending services for some years and gave enduring consent for the research use of what remained of their samples, which would otherwise be thrown away.6 One explained, ‘I wouldn’t be here today if it weren’t for the women who came before and the people who tried to keep them alive. They gave their breast tissue and bloods for research, and they tried out so many different treatments.’. Research participation thickened the textures of waiting *with* other people and imagining the afterlives explicitly conceived in experimental cancer care to which participating patients—and staff—will belong only indirectly.7

Smith wanted to find traces of her contributions so that she could imagine an afterlife in which she could recognise herself and perhaps win recognition. Research findings reported to participants were anonymised and they rarely elicited affinities in the way of a negotiated ‘written portrait’: it was hard to like a representation that aggregated materials from innumerable others across many centres in the form of statistical correlations that you could not recognise as a likeness. But Smith collaborated with Day and Ward to track some of her contributions to the hospital and university tissue collections, data resources and clinical trials. Despite what seemed a disappointing lack of closure when Day and Ward were unable to report any definitive findings, Smith concluded,

The process of contributing to research is a positive incentive, and makes you feel a bit more special and supported. However, don't be under any illusions that your contribution will, on its own, be responsible for any “Eureka” moment - it is still an unidentifiable drop in the ocean. But without all the drops there would be no ocean. (cited inDay, Smith, and Ward in press)

We drew (above) on Bourdieu’s concept of affinity or liking—‘taste’—from which categories of people can be inferred. Other concepts of affinity are significant for understanding their affective purchase, including Mason’s consideration of affinity-for-itself (Mason 2018). This suggests the vitality or atmospheric charge that made some of the perspectives adopted in waiting and participating in personalisation more potent than others. For example, Jayne Smith and other women we knew were aware of a popular rhetoric in the UK that pits generations against each other in a kind of actuarial accounting and associates seniors with unfair privileges. In popular and academic accounts, it is often younger not older people today who are considered to be waiting involuntarily for their lives to begin again, victims of wider forces, left out and forgotten. A generational public ledger effectively naturalises historical processes that have dismantled Keynesian relations between state, market and family. Unsurprisingly, these senior patients, most of whom were retired, disputed the naturalisation.

Patients learnt from staff about the importance of their contributions for subsequent patients, a situation echoed in the comment cited above from a woman who did not think she would be alive but for the assistance of previous generations. Such contributions are viewed both as donations and as expropriations, that is, samples extracted without compensation (Cooper and Waldby 2014; Pinel and Svendsen 2021). We consider that they can be accommodated to a sense of waiting on the part of patients attending services over several years. This waiting established relational, comparative and perspectival scaling through what Gal and Irvine call fractal recursion (above; see also Gal 2016; Irvine 2016; Irvine and Gal 2000); women reapplied comparisons of waiting with cancer to other situations, such as a more encompassing comparison of generations. In our interpretation, this comparison suggests that elders, including those without families, make key contributions to social reproduction even though these may only be delineated clearly in retrospect. Waiting of this kind anticipates a return that is indirect rather than direct but still creates a sense of belonging.

This interpretation of generational relations echoes Marcel Mauss’ well-known essay on the gift a century ago. When it went to press in 1925, the essay was explicitly listed as one of two memorials, prefaced by an account of those who had died during World War I, including a frontispiece with a photograph of his uncle, Emile Durkheim. The few essay pages are sandwiched between an eulogy to those who had died since 1912 and an extensive set of reviews. Jane Guyer, in her introduction to a 2016 edition, reflects on Mauss’ exploration of Jewish funerary ritual that is described in English as ‘the true gift’ (Mauss 2016). The relevant Hebrew terms are translated as steadfast remembering, compassion and the saving of life. A gift of life can never be assimilated to other gifts, Guyer emphasises, but it might perhaps be returned indirectly across generations. This reading is strengthened by Mauss’ connections with solidarism. Politicians such as Léon Bourgeois (1851–1925) saw a quasi-contract among present and future generations who remained continuously indebted to previous members of society (Narotzky 2021). Mauss’ essay suggests that the past persists or recurs; the gift—perhaps also a sacrifice—can be recuperated as a mode of repair.

In 2020, women living with cancer all felt excluded at times and full of anxiety as they tried to understand and follow confusing shielding guidelines, waiting to find out too whether their cancers would wait with them as health services were reorganised. One of the long-standing patients we have known for several years described unanticipated benefits in the first months of COVID-19, ‘Now, other people know what it’s like [that is, waiting] and I don’t have to explain all the time’. Since everyone had placed elements of their lives on hold, she felt less stigmatised by a condition that she had to constantly explain or conceal. Increased risk of infection was attributed to older age groups and so seniority—regardless of cancer status—became a critical axis of differentiation in waiting, especially during the first wave of the pandemic in the UK.8

This summary illustrates the multiple and shifting perspectives developed by participants as they positioned themselves relationally and comparatively inside and outside ‘the picture’, scaling the molecular with the human across generations (Barad 2003; Summerson Carr and Lempert 2016).9 Patients drew Maggie’s and the hospital into varied relations that included snapshots from *Written Portraits* and the years that continued to unfold in an experimental care available to everyone in principle but organised through complex queues informed by different priorities and possibilities.

Participation in the poetry residency can be framed by the ‘cutting’ of a bundle of property rights when a liking (affinity) coincides with a likeness (resemblance). It was at this point that Sherlock saw herself as author of the finished portrait which she co-owned with sitters. Participation in experimental cancer care by contrast involved repeated cuts to this bundle from the time consent was given, during subsequent care and in continued reuse of ‘pictures’ made from patient data and tissue.10 These might become resources for testing and retesting scientific and clinical categories, devices and treatments for a personalised cancer medicine, or indeed unrelated research studies including training new researchers and new devices.

These cuts make different genres of property as well as pictures from the traces of participation. We have emphasised intersubjective qualities in the recognition exchanged with the liking for and of a likeness. From Sherlock’s perspective, the giving back of a poem worked best when it achieved the ‘honouring’ that a sitter described from a previous residency, which was echoed in an email after the online launch of *Written Portraits*. A sitter wrote,

I was reluctant to share the mirror you held up to me until now. I thought it might be too self-obsessed, esp. as I forgot to mention the cancer…!” My father, a Scotsman, was fond of quoting Burns and there’s a few lines that stuck in my head;

O wad some Power the giftie gie us

To see oursels as ithers see us!

It took a while for that “portrait” to percolate through, and to accept this “gift”… [which] also requires acceptance and perhaps the realisation that we're not nearly as self-aware or as distant from a Trumpian view as we might like…

At the launch he had explained, as others did, that the poet was a very good listener and observer. He continued, ‘[she] held a mirror to me that allowed me to see myself perhaps as others do and not as I had imagined myself. Oddly enough I’d missed out the cancer bit even though it has profoundly changed the way I experience the world’ (https://peoplelikeyou.ac.uk/activities/book-launch-written-portraits-by-di-sherlock/).

As we have shown, the gifts or returns from experimental care are likely to be deferred and indirect. Although Smith considered that a clinical trial in which she had participated also saved her life, her comments show that the links between a research donation and the uses to which it is put are generally obscured by property relations in the hospital. Davina Cooper has argued that a subject-object relation of property requires disconnection, as in most hospital research (Cooper 2007). By contrast, a part-whole relation requires connection of the kind transacted in the poetry residency. Cooper suggested that this second understanding informed an understanding of property as ‘a set of networked relations in which the subject is embedded rather than … simply exercising mastery or control over an object’ (Cooper 2007, 636). These contrasting property relations often combine, as Cooper showed with reference to the variegated social of Summerhill School. Similarly, although patients’ research contributions may have been donated or taken without compensation, we suggest that they also forge connections that embed participants in a collective. For example, Smith put her research contributions, which had ‘disappeared anonymously into an abyss of data, together with those of millions of other cancer patients - just a drop in the ocean’ (cited in Day, Smith, and Ward in press) into her own perspective on belonging. Drawing on our work with Smith and other women living with breast cancer, we find that traces of participation combine and circulate. Contributions are reconfigured when a poem is edited, and the collection circulates for different uses; belonging turns into belongings and belongings create a sense of belonging to and being ‘in the picture’ as they are scaled. With time, different ‘cuts’ to bundles of property rights leave residues that also accumulate and can sometimes be transformed into assets and resources for public and private use.

## Conclusion

It is common to differentiate the creative or artistic from (other forms of) research. We hope that our reflections will contribute to further discussion in the medical humanities on what participation does in and across distinctive genres of enquiry. In our broader collaborative project on personalisation, we have been interested in practices that make different and overlapping categories of ‘people like you’—in English, ‘you’ is both singular and plural. A mode of address, we suggest, invites and formats *participation* to collect relevant data. Aware of our contemporary ‘participatory condition’ (Barney *et al*. 2016), we have asked what three different kinds or genres of participation might do in the context of cancer care. Genres invite, address and presuppose an audience; they are also combined, circulated and changed by their audiences.

Category-making in cancer medicine, as in many areas of life today, is enabled if not dependent on increasingly intensive, rapid computation of extensive data on behaviours and activities which are typically combined with long-established records of vital statistics and sociodemographic attributes. In cancer medicine, what participants like and resemble is analysed to produce *precise* classifications which are tested and retested for their *predictive* utility (Day, Lury, and Ward in press). Personalised medicine is understood to focus on synchronised matching of biological markers with disease through analysis of clinical, biological and conversational data. These enable the construction of types of cancer with affinities for certain treatments, that is, ‘people like you who like things like this’. Patients were classified for the purpose of treatment by several kinds, grades and stages of breast cancer and predictions about their development. A patient’s provisional type was updated periodically according to the evolution of her cancer, including resistance to treatment, and new evidence.

Because of such technical developments, many personalising practices seem far removed from mundane processes of interaction. But biological markers were not the only ones that mattered. We categorise ourselves and others routinely according to what we like and resemble. We have shown that participants in public science cafés and a poetry residency also registered and categorised—or pictured—affinities and resemblances for varied purposes. Data from conversations with the poet were ‘formatted’ to distill and illuminate experiences that represented a person or a group in a portrait. Sitters and potential sitters were able to recognise the people in or behind these portraits and, in recitals from and discussions of *Written Portraits*, they valued this individual and collective ‘honouring’.11 Participants in science cafés also selected and compared varied associations for their relevance as they debated ‘better’ ways of understanding and treating cancer, and implementing them, and acknowledged the significance of different positions and priorities. Material from these public meetings informed recruitment to further, more formal participation and the development of cafés for other purposes. Traces of participation were thus registered in creative practice and public discussions about personalisation that were also regularly adjusted and updated.

Participants working and living with cancer drew on mundane associations to interpret the technical categories of personalised cancer medicine. Moreover, they exchanged perspectives and our discussion of long-standing patients shows how they put themselves inside as well as outside a picture through fractal comparison of different aspects of waiting that combined data from personalised cancer medicine, conversations and other forms of participation. With time, these patients recognised themselves in relation to the typing of cancers and, evaluating their position, simultaneously assembled provisional guides for navigating the environment. Exchanging perspectives, they too created shifting categories of ‘person’ from ‘relevant’ data assembled for different uses. Some perspectives exclude those depicted from recognition or use; others construct property relations that provide the means to navigate cancer care and create a sense of belonging. ‘In the picture’, these women anticipated returns in the form of continuing care, a poem, possible futures and/or recognition.

‘Like’ is a small word to inspire this classificatory imagination as ‘Johnson’ writes ‘in defence of a little word with many critics and lots of uses’ in an Economist article (‘Johnson’ 2021). They suggest, ‘if you, like, dislike “like”, maybe, like, think again’ and cite the linguist Lawrence Schourup (1983) who wrote that like ‘is used to express a possible unspecified minor nonequivalence of what is said and what is meant’. If Sherlock aims for an equivalence between a likeness and a liking that will capture a transaction over a relatively short time frame, she and her sitters also acknowledge the non-equivalence that is so apparent in the ‘likes’ punctuating emerging cancer pathways to associate equivocally one with another, you singular with you plural, and individual and collective.

## Data Availability

Data may be obtained from a third party and are not publicly available.

## Notes

1. We would like to acknowledge the 2020–2021 cohort of Masters’ students at Imperial College London for their contributions. We would also like to note that this article is ‘led’ by questions about participating in personalisation. This amplifies a collective authorial position in which the poet’s own distinctive voice is described at a distance through another genre. Di Sherlock, like her sitters, came to see herself, or rather her practice, differently in the way others describe and see it (see pp.4–5). Sherlock added, at the time of writing, how this process ‘continues to unfold as my voice is further voiced by actors in performance. The recitation, for me, generates further insight into the practice both by hearing my words animated by another and by listening to the thoughts the recitation provokes in the sitters and general listeners’.

2. They elaborated these topoi as, ‘the suffering patient, a doctor’s practice of clinical care, the exemplary site of the clinic, and cancer – as congealed, and overly resonant configurations that constitute but one particular way of making phenomena come to matter’ (2016: 41)

3. Further details on our methods are described in Day *et al*. (2021); see also Viney *et al*. (2022).

4. This individualisation belongs as much to formal as to informal ethics, including those associated with consent to participate in hospital research.

5. An exhibition at Christie’s London of the portraits can be viewed online at: https://zoelaw.com/exhibitions/life/.

6. Enduring consent is now routinely requested in this setting for sharing any excess from samples obtained in usual care.

7. Although we focus on patients, staff too are expected to participate in or facilitate authorised university and NHS research.

8. The primary referents that women made were to historically situated or familial cohorts of people but ‘generation’ merits more consideration than we can provide here. For example, generational positioning is no doubt modulated by gender, among other factors, but we can only speculate about the relevant contours since this part of our work was conducted exclusively with women. It is also worth noting that references were made to ‘next generation’ or ‘poster child’ techniques, tests and treatments in the hospital, the use of which also stratified women who were otherwise considered contemporaries into distinct cohorts.

9. In her subsequent edition of *Partial Connections*, Strathern defines scale as ‘the organisation of perspectives on objects of knowledge and enquiry’ (Strathern 2005).

10. See Pottage and Marris on the complexities of anticipating property forms—or the ‘cut’ that makes a ‘part’—in synthetic biology (Pottage and Marris 2012).

11. This sense of recognition and honouring evokes affinities of the kind Jennifer Mason (2018) delineates.
